# Trust and its relations to vaccine beliefs: a latent class analysis on 140,000 individuals worldwide

**DOI:** 10.1093/eurpub/ckac131.389

**Published:** 2022-10-25

**Authors:** F Sanmarchi, D Gibertoni, D Golinelli, D Gori, LM Scheier

**Affiliations:** 1 Department of Biomedical and Neuromotor Science, Alma Mater Studiorum - Università di Bologna, Bologna, Italy; 2 Research and Innovation Unit, IRCCS Azienda Ospedaliero-Universitaria di Bologna, Bologna, Italy; 3 LARS Research Institute, Inc., Scottsdale, USA

## Abstract

**Background:**

Research shows that vaccine-related beliefs (i.e., about efficacy, safety, purpose) may reflect a host of within-person and contextual factors yielding homogeneous subgroups of individuals. This study aims to characterize distinct subgroups of people and identify ideal targets for tailored public health interventions to increase vaccine adherence.

**Methods:**

Latent class analysis was used to derive subgroups based on unique response profiles using the 2019 Gallup survey of 140 countries (>140,000 individuals). We modeled a composite of vaccine beliefs as a distal outcome examining differences for the obtained classes, with and without covariates in the model.

**Results:**

A 5-class model fit best with classes distinguished primarily on whether individuals possessed or sought personal knowledge about science, medicine, and health, whether they trusted science, scientists and have confidence in the healthcare system. The lowest levels of vaccine beliefs were reported by a class not endorsing any of these indicators and the highest levels by a class endorsing all the indicators (p < 0.001). Age class showed a U-shaped relation with vaccine beliefs, while higher educational level (p = 0.025), higher subjective income (p = 0.006) and employment (p < 0.001) were related to higher vaccine beliefs. Country-level income was moderately related to class membership and vaccine beliefs were higher in lower-income countries (p < 0.001).

**Conclusions:**

Our findings suggest that more work is needed to improve trust in science and medical providers. Tailored interventions grounded in a community-based and empowering approach with the collaboration of multiple stakeholders seems to be needed to improve vaccination rates. This can only be achieved when individuals trust science, scientists and healthcare providers and accrue the necessary wisdom to make good healthcare decisions that affect not only themselves but their fellow citizens.

**Key messages:**

• Efforts to alter vaccine beliefs should touch on where people access information on science and health, the processes that build trust, and their belief whether science improves well-being.

• Public health interventions should focus on reassuring individuals that science and health workers are benevolent. An essential first step in the health worker-patient relations is building trust.

